# Physical health and activity management in forensic mental healthcare: hospital practices and staff insights from an Australian national study

**DOI:** 10.1192/bjo.2026.12013

**Published:** 2026-06-02

**Authors:** Katherine Moss, Carla Meurk, Megan L. Steele, Ed Heffernan

**Affiliations:** School of Public Health, University of Queensland, Brisbane, Australia; Forensic Mental Health Research Stream, Queensland Centre for Mental Health Research, West Moreton Hospital and Health Service, Brisbane, Australia; Queensland Forensic Mental Health Service, Metro North Hospital and Health Service, Queensland Health, Brisbane, Australia

**Keywords:** Physical health, physical activity, exercise, forensic psychiatry, nutrition

## Abstract

**Background:**

There is currently no national approach to physical health and activity management for individuals under forensic mental healthcare.

**Aims:**

This study aims to document the physical health and activity management of in-patients under forensic mental healthcare across Australia, physical activity opportunities and how data are recorded. It also explores staff perspectives on physical health and activity needs of forensic in-patients.

**Method:**

Forensic hospital staff with expertise in managing the physical health and activity of in-patients were invited to participate in a mixed-methods study.

**Results:**

Twenty-two surveys were completed and 64 staff members participated in 17 focus groups. Eight themes were identified: (a) lack of a standardised approach to physical health and activity; (b) inconsistent monitoring and documentation; (c) importance of general practitioners and multidisciplinary teams; (d) need for structured, adaptable programmes and engaged staff; (e) challenges related to patients, staff, ethics and infrastructure; (f) gaps in education, engagement and awareness; (g) need to balance nutrition and eating autonomy; and (h) importance of developing and implementing interventions.

**Conclusions:**

Forensic hospitals in Australia lack a standardised approach to physical health and activity management, with inconsistent monitoring. Physical activity interventions require engaged staff, resources and flexibility, yet ethical and logistical barriers complicate implementation. Solutions include investing in specialised staff, scheduling physical activity into patients’ routines and integrating information technology systems. Comprehensive policies for assessment, monitoring, nutrition, physical activity and interventions are important for improving in-patient forensic mental healthcare, and key performance indicators should be developed.

Forensic mental healthcare in Australia is provided in secure hospitals that have higher levels of physical, relational and procedural security than general mental health hospitals. The patient cohort includes three groups: those on a court-based order who have been found, because of their mental illness, to be either not criminally responsible of an offence or unfit for trial; those who have been transferred for in-patient mental healthcare from the criminal justice system; and those who have problematic behaviours that cannot be effectively managed in general mental health wards. Forensic patients are generally diagnosed with chronic psychotic illnesses such as schizophrenia, and are treated with medications that have a range of side-effects including weight gain. Across Australia there are seven forensic hospitals with varying levels of security (low, medium and high). These forensic hospitals play a critical role within the broader mental health system by providing secure and specialised care for these individuals that pose risks to the community, thereby protecting community safety while supporting rehabilitation and recovery. Although these hospitals provide important interventions for patients, they have been criticised for lacking consistent standards of care, neglecting human rights and relying on insufficient quality evidence for interventions.^1^

Australia’s National Mental Health Commission has highlighted that equity in health is a fundamental human right.^2^ Although there is no national Human Rights Act, the Australian Capital Territory, Victoria and Queensland have all passed their own state and territory legislation.^3^ In light of human rights principles, it is important to highlight that forensic patients are entitled to equivalency of care, that is, healthcare of the same standard and quality as that available to individuals in the broader community. The challenge is that forensic hospitals are highly structured environments. Strict security protocols govern access into the hospital environment. Access to hospital wards is also tightly controlled.

There is already a significant life expectancy gap of at least 11 years for those who access mental health-related treatments compared with the rest of the population.^4^ Older estimates have reported a gap of up to 20 years for those with severe mental illness.^5^ Cardiovascular disease and poor physical health are contributing factors. Research considering forensic in-patient populations, frequently subject to long in-patient stays, has highlighted the risks of being overweight/obese, gaining weight during admissions and developing metabolic syndrome and associated medical illnesses.^6,7^ Further, forensic in-patients have reported low levels of physical activity,^8^ putting them at increased risk of a range of conditions, including cardiometabolic disorder, which increases the risk of premature death.^9^ In a Queensland cohort, patients’ length of stay ranged from 55 to 6852 days (median 741 days, interquartile range 1322.5), with some patients staying up to 18.8 years, illustrating the prolonged duration of forensic in-patient care.^8^

Evidence from forensic populations highlights the severity of these risks. Rees and Thomson^10^ followed a Scottish forensic cohort for a mean of 21.1 years and found that 36.9% of patients died at an average age of 55.6 years, with 70.8% of deaths occurring prematurely. Men lost on average 14.9 years and women 24.1 years of potential life. These findings underscore the critical need for forensic hospitals to have a structured and comprehensive approach to physical health management, with strategies in place for screening and monitoring physical health.

There is widespread recognition of the need for effective intervention strategies to address health screening findings.^11,12^ Lifestyle interventions targeting diet, physical activity and behaviour modification have been shown to improve body weight, fitness, psychiatric symptoms and physical activity levels.^13,14^ However, research on the effectiveness of these interventions in forensic populations remains limited.^15,16^ Although some studies in forensic settings have explored approaches including technology, education and exercise, they lack randomised control groups and do not consistently report on physical health outcomes, leaving a significant gap in the literature.

Emerging literature highlights the perspectives of both patients and staff on physical health and activity in forensic hospitals. Studies have explored how adolescent and adult in-patients perceive the role and significance of physical activity, as well as their experiences and preferences. Anthony and colleagues found widespread agreement among patients and staff that physical activity offers multiple benefits, including improved mood, increased engagement and enhanced pro-social behaviours.^17^ They concluded that health promotion and physical health support should be integrated into service provision, with staff collaboration being essential. Similarly, the authors of this current study recently emphasised the importance of social and environmental factors in encouraging physical activity and further supported its inclusion within a comprehensive treatment framework.^18^

Given gaps in physical healthcare for forensic in-patients, and government undertakings at both state and federal levels to better support the physical health needs of mental health consumers, a national examination is timely.^19,20^ Currently, there is no national approach to assessing and monitoring physical health or providing physical activity opportunities for forensic in-patients. This study had three aims:

- to examine the physical health and activity data collected by staff at forensic hospitals;
- to evaluate how physical health issues are managed, the available physical activity opportunities, and who supervises, prescribes and monitors these activities;
- to assess staff perspectives on facilitators and challenges of addressing the physical health and activity needs of forensic in-patients.

## Method

### Context

The setting for this national study included the forensic hospitals across Australia, comprising the High Secure Inpatient Service in Queensland, Forensic Hospital in New South Wales, Thomas Embling Hospital in Victoria, James Nash House in South Australia, Frankland Centre in Western Australia, Dhulwa Secure Mental Health Unit in the Australian Capital Territory and Wilfred Lopes Centre in Tasmania.

### Study design

A mixed-methods study was undertaken, including an online survey and focus groups. The conduct and reporting of the qualitative elements of the study adhere to the Standards for Reporting Qualitative Research.^21^

### Ethics

The authors assert that all procedures contributing to this work comply with the ethical standards of the relevant national and institutional committees on human experimentation and with the Helsinki Declaration of 1975, as revised in 2013. All procedures involving human patients were approved by the Royal Brisbane and Women’s Hospital Research Ethics Committee (HREC/2021/QRBW/78458) and ratified by the University of Queensland. It was applied for under the multi-site National Mutual Acceptance scheme. Seven sites were included in the application including the High Secure Inpatient Service in Queensland, Forensic Hospital in New South Wales, Thomas Embling Hospital in Victoria, James Nash House in South Australia, Frankland Centre in Western Australia, Dhulwa Secure Mental Health Unit in the Australian Capital Territory and Wilfred Lopes Centre in Tasmania.

Informed consent (either written or verbal) was obtained for all participants. Verbal consent was witnessed and formally recorded.

### Sample and data collection

The clinical directors of each forensic hospital were contacted by the investigators and informed of the study. The clinical directors then nominated key stakeholders (senior staff members including psychiatrists, nurse unit managers and allied health team leaders) to assist in the identification of participants. Participants were eligible if they had expertise in the prescription, supervision and/or management of physical health and activity of forensic in-patients and had worked within a forensic hospital for more than 6 months. The investigators were then provided with the email addresses of potential participants who were emailed and invited to take part in the study.

The online survey was completed by participants who consented to the study, via a link that was sent by email to participants. Focus groups were organised by the principal investigator (K.M.) and the site coordinators at each of the forensic hospitals. Participation in the survey and focus groups was independent, as the survey was anonymous apart from state/territory and occupation. Therefore, overlap between survey and focus group participants cannot be determined, although some participants may have contributed to both. The number of participants per hospital in each data source was as follows: survey participants (Queensland: 6, New South Wales: 3, Victoria: 5, South Australia: 2, Western Australia: 2, Australian Capital Territory: 3, Tasmania: 1; *n* = 22 total); and focus group participants (Queensland: 10, New South Wales: 8, Victoria: 11, South Australia: 13, Western Australia: 6, Australian Capital Territory: 8, Tasmania: 8; *n* = 64 total).

Of the 17 focus groups conducted, 16 were held via Microsoft Teams and recorded and transcribed using the platform, whereas one was conducted face-to-face. Group size varied because of participant availability, but data were analysed at the state/territory level, supporting thematic saturation within and across jurisdictions. Data collection occurred between September 2023 and January 2025.

Both the survey and focus group schedule were developed by K.M., C.M. and M.L.S., based on the Royal Australian and New Zealand College of Psychiatry’s clinical practice guidelines for schizophrenia and related disorders and the expert consensus statement for the treatment, management and monitoring of the physical health of people with an enduring psychotic illness.^22,23^ Both the online survey and interview script are available in the Supplementary Material available at https://doi.org/10.1192/bjo.2026.12013.

### Survey

The survey was created in Checkbox (2023 release, web-based platform, Checkbox Technology Inc., San Francisco, California, USA; https://www.checkbox.com), an online survey platform, and comprised questions regarding the following:

- health measures on initial assessment and monitoring, and documentation;
- physical activity measures, opportunities and data tracking;
- staffing and support available;
- facilities and equipment available;
- patient and carer education and involvement.

### Focus group

The focus group included questions posed in the online survey and covered the following topics:

- physical health assessment and monitoring;
- patient and carer engagement;
- data collected regarding physical health and/or activity measures and lifestyle interventions;
- physical activity and obesity management;
- adherence to guidelines and best practice;
- facilitators and barriers.

### Data analysis

The study utilised a fully mixed concurrent equal status design, integrating qualitative and quantitative data collected simultaneously through an online survey and focus groups.^24^ Both types of data were analysed independently before being integrated for comprehensive interpretation.

Responses to the survey were automatically recorded in Checkbox. The responses were also exported to Microsoft Excel and manually reviewed. Of the 32 survey questions completed by 22 participants, 63 responses were missing across 25 questions. Missing data were handled on a per-question basis: for descriptive analyses, available responses were used to calculate totals and percentages, without imputing missing values. Given that multiple respondents contributed information from each hospital, missing responses were assumed to reflect gaps in individual knowledge rather than systematic non-response bias. This approach allowed hospital-level patterns to be reliably assessed despite occasional missing data.

Challenges arose for consistent data representation when staff working within the same facility provided varied responses to survey questions. To address this, it was decided that the presence of a particular practice or resource would be accepted if at least one staff member endorsed it. This approach was based on the understanding that not all staff would be equally informed about all aspects of service provision, and that endorsement by a single respondent was more likely to reflect accurate knowledge based on their specific expertise or role.

The responses from the online survey and focus groups were analysed using an established thematic framework method.^25^ One researcher (K.M.) performed the coding and analysis, following the five stages of the method. The first stage involved becoming familiar with the data by reading and rereading it, noting down initial observations. In the second stage, data were coded using both inductive and deductive approaches. The initial coding was conducted in Microsoft Excel. The subsequent stages focused on grouping the codes into potential themes, which were visually represented on a thematic map to aid in the process. This helped to identify broad initial themes and their related subthemes. A final review of the themes ensured clarity and distinctiveness between them. The finalised themes and sub-themes were discussed and agreed upon by all study authors, and representative excerpts were selected for inclusion.

The identified themes will structure the presentation of findings from the study.

## Results

A total of 22 surveys were completed between March 2023 and January 2025. There was a range of experience for survey participants working in a forensic hospital environment, from 0.5 to 15 years, with a mean of 5.0 years (s.d. 3.5). Sixty-four individuals participated in 17 focus groups between September 2023 and November 2024. Participant demographic data are shown in Table 1. Survey and focus group participants were broadly similar, and comprised senior clinical staff such as psychiatrists, nurses, occupational therapists, allied health practitioners, and other professionals involved in physical health and activity management (Table 1). Focus groups explored the same thematic areas as the survey, but in a qualitative, discussion-based format. Data from surveys and focus groups were combined at the state/territory level in Tables 3 to 5 to provide a more complete view of staff perspectives while maintaining confidentiality for sites with very small survey numbers.

**Table 1:** Demographics of participants

Demographic	Survey participants	Focus group participants
	(*n* = 22), *n* (%)	(*n* = 64), *n* (%)
State		
Queensland	6 (27.3)	10 (15.6)
Victoria	5 (22.7)	11 (17.2)
New South Wales	11 (50.0)^a^	8 (12.5)
South Australia		13 (20.3)
Western Australia		6 (9.4)
Australian Capital Territory		8 (12.5)
Tasmania		8 (12.5)
Occupation		
Doctor	7 (31.8)	13 (20.3)
Allied health	7 (31.8)	22 (34.4)
Other	8 (36.4)	3 (4.7)
Nurse		13 (20.3)
Allied health assistant/diversional therapist/recreation officer		8 (12.5)
Exercise physiologist		5 (7.8)

a. Combined across New South Wales, South Australia, Western Australia, Australian Capital Territory and Tasmania.

**Table 2:** Physical health and activity measures on admission and monitoring in forensic hospitals

Measure	Admission	Monitoring	Measure	Admission	Monitoring
	*n* (%)	*n* (%)		*n* (%)	*n* (%)
Weight	13 (59.1)	10 (45.5)	Cholesterol	8 (36.4)	9 (40.9)
Height	12 (54.5)	15 (68.2)	Fasting insulin	1 (4.5)	1 (4.5)
Body mass index	12 (54.5)	7 (31.8)	FBC	11 (50.0)	7 (31.8)
Waist circumference	7 (31.8)	8 (36.4)	eLFTs	9 (40.9)	8 (36.4)
Blood pressure	15 (68.2)	13 (59.1)	TFT	4 (18.2)	6 (27.3)
Resting heart rate	13 (59.1)	10 (45.5)	Hepatitis screen	2 (9.1)	3 (13.6)
Electrocardiogram	9 (40.9)	8 (36.4)	HIV screen	2 (9.1)	2 (9.1)
Haemoglobin A1c	7 (31.8)	7 (31.8)	Smoking	8 (36.4)	5 (22.7)
Fasting blood glucose level	7 (31.8)	7 (31.8)	Diet	4 (18.2)	6 (27.3)
Two separate fasting blood glucose levels	2 (9.1)	2 (9.1)	Physical activity history	2 (9.1)	4 (18.2)
Triglycerides	7 (31.8)	9 (40.9)	Other	2 (9.1)	3 (13.6)
HDL-C	6 (27.3)	8 (36.4)	No answer/I don’t know	6 (27.3)	6 (27.3)
LDL-C	6 (27.3)	8 (36.4)	Physical activity measure^a^	2 (9.1)	2 (9.1)
Survey and focus group results					
Theme: Lack of a standardised approach to physical health and activity					
Subthemes	Quotes – survey participant (P) and focus group (FG)				
Inconsistent responsibility and structure	‘We could be more prescriptive in both the systemic and individual level for physical health monitoring…we could probably have some procedure in in place monitoring’ (FG8) ‘There is a feeling that there could be more strategy and more leadership in terms of our physical health approach’ (FG11)				
Variable measures and data collection	‘Some of the measures are collected by the exercise physiologist however the specific measures depend on the particular exercise physiologist’ (P3) ‘I think we measure BMI [body mass index]… there isn’t a standardised set of bloods we do’ (FG4)				
Limited activity level assessments	‘I don’t think there’s any formal specific, you know, assessment of physical activity levels or fitness’ (FG3) ‘Skin folds…we can do all that stuff if they want it…6-minute walk test, sub-max Treadmill test…they need to want that otherwise it’s not worthwhile’ (FG12)				

FBC, full blood count; eLFTs, electrolytes and liver function tests; TFT, thyroid function tests; HDL-C, high-density lipoprotein cholesterol; LDL-C, low-density lipoprotein cholesterol.

a. Six-minute walk test, the International Physical Activity Questionnaire and/or Simple Physical Activity Questionnaire.

**Table 3:** Metabolic monitoring, documentation and data collation practices in forensic hospitals

State		Electronic record for metabolic monitoring	Paper chart for metabolic monitoring	Specific metabolic monitoring form	Previous collation of physical health data
Queensland		X	–	X	X
New South Wales		X	–	X	X
Victoria		X	X	X	X
South Australia		X	X	–	–
Western Australia		X	X	–	–
Australian Capital Territory		X	–	X	–
Tasmania		X	X	–	–
Survey and focus group results					
Theme: Inconsistent monitoring and documentation					
Subthemes	Quotes – survey participant (P) and focus group (FG)				
Uncertainty about current practices	‘I’m unsure what physical health data is collected’ (P17) ‘I think we just put it on, probably correct me if I’m wrong…I think it’s all entered on just the overall database…I’m not sure if there’s something separate to that…could be wrong though’ (FG9)				
Inconsistency and challenges with documentation	‘Our documentation system is like a black hole when it comes to putting information in, you put it in, but you can’t generate a report out’ (FG8) ‘There is no single database that is uniform throughout the hospital’ (FG9) ‘Poor integration of bloods and metabolic data…a lot of double handling’ (FG15)				
Discontinuation of structured data collection	‘Tried a couple of ways over the years…pre and post kind of assessments, either of knowledge or motivation as well as BMI… that was probably about 8 years ago, and that was probably the most structured’ (FG6) ‘Used to check BMI and graph it, previous dietitian did that, helpful to a point’ (FG12)				

X, endorsed by at least one participant from forensic hospital in state/territory; –, no survey participants from the site endorsed this response; BMI, body mass index.

**Table 4:** Staff providing support for physical health assessments and physical health interventions

Survey results											
	Staff providing support for physical health assessments										
State	Psychiatrist/registrar	General practitioner/medical officer	Nursing staff	Physiotherapist	Occupational therapist	Exercise physiologist	Personal trainer	Diversional therapist/recreational officer	Dietitian	Allied health assistant	Other
Queensland	X	X	X	X	–	–	–	–	–	–	–
New South Wales	X	X	X	X	X	X	–	–	X	–	
Victoria	X	X	X	–	–	–	–	–	–	–	Nurse practitioner
South Australia	X	X	X	–	–	–	–	–	–	–	–
Western Australia	X	X	X	–	–	–	–	–	–	–	–
Australian Capital Territory	X	X	X	–	–	X	–	–	–	–	–
Tasmania	–	–	X	–	–	–	–	–	–	–	–

	Staff providing support for physical health interventions										
State	Psychiatrist/registrar	General practitioner/medical officer	Nursing staff	Physiotherapist	Occupational therapist	Exercise physiologist	Personal trainer	Diversional therapist/recreational officer	Dietitian	Allied health assistant	Other
Queensland	X	X	X	X	X	–	X	X	–	–	–
New South Wales	–	–	X	X	X	X	–	X	–	X	–
Victoria	X	X	X	X	X	X	X	–	–	–	Gym staff
South Australia	–	X	X	X	X	–	–	–	–	X	Psychologist
Western Australia	X	X	X	–	X	X	–	–	X	–	–
Australian Capital Territory	X	X	X	–	X	X	–	–	–	X	–
Tasmania	–	–	X	–	X	–	–	–	–	X	–
Survey and focus group results											
Theme: The importance of GPs and MDTs											
Subthemes		Quotes – focus group (FG)									
The MDT collaborates to support patients’ physical health and activity		‘A lot of the walking tracks, the gym, getting people into the gym and then also getting people to groups like volleyball and badminton that’s really a shared thing between allied health and nursing’ (FG6) ‘Now EP and OT and AHAs can facilitate this so don’t need to cancel anything…now it’s a well-run programme, everyone works well together, same vision’ (FG12)									
Access to GP services		‘GP does a brilliant job, but…we could use more’ (FG10) ‘We’ve got access to a GP here, which is really good…it would be good if there was increased time and increased access to the GP service because they are, I think stretched’ (FG11)									
Need for specialist exercise physiologists		‘We can provide opportunities…but…it’s not as effective as someone like an exercise physiologist who can write a programme and tailor it to that person’ (FG6) ‘Having an exercise physiologist is important…someone who is attune to mental health needs’ (FG15)									
MDT meeting to review physical health and activity		‘Access to the gym needs to be approved through the MDT process…there’s consideration of suitability for exercise’ (FG7) ‘We meet twice a week, and we discuss certain patients…physical health is discussed there, it is not necessarily something that is prioritised and a lot of the time it can be missed unless members of the MDT are present to sort of raise that’ (FG9)									

X, endorsed by at least one participant from forensic hospital in state/territory; –, no survey participants from the site endorsed this response. GP, general practitioner; MDT, multidisciplinary team; EP, exercist physiologist; OT, occupational therapist; AHA, allied health assistant.

**Table 5:** Physical activity environments, equipment and opportunities for patients under forensic mental healthcare

		Physical activity environments and equipment										
State		Gym	Outdoor exercise equipment	Tennis court	Swimming pool	Equipment on wards	Treadmill		Rowing machine	Other		
Queensland		X	X	X	X	X	X		X	Basketball court, gym equipment, bikes		
New South Wales		X	X	–	X	X	X		X			
Victoria		X	X	–	X	–	X		–	Weights machine		
South Australia		X	X	–	–	X	X		–	Exercise bike		
Western Australia		X	X	–	–	X	X		X	Community based exercise activities (e.g. swimming and squash)		
Australian Capital Territory		X	X	X	–	X	X		X	Fixed weights machine, outdoor walking track, half-sized basketball court, exercise bike, bikes		
Tasmania		X	–	–	–	–	-		X	Exercise bike		

	Physical activity opportunities offered to patients									
State	Individual programme on ward	Individual programme at gym	Group programme on ward	Group programme at gym	Swimming	Tennis	Soccer	Volleyball	Walking group	Other
Queensland	X	X	X	X	X	X	X	X	X	Bike, gardening
New South Wales	X	X	X	X	X	–	X	X	X	
Victoria	X	X	X	X	X	X	X	X	X	Yoga, water aerobics, circuit training
South Australia	X	X	X	X	-	X	–	X	X	Badminton
Western Australia	X	X	–	X	X	–	X	X	X	Squash, exercise circuit
Australian Capital Territory	X	X	X	–	X	X	X	X	X	Fishing, badminton groups, swimming, golf, street soccer, biking
Tasmania	–	–	–	–	–	–	–	X	X	Circuit class
Focus group results										
Theme: The need for structured, adaptable programmes and engaged staff										
Subthemes		Quotes – focus group (FG)								
Physical activity through structured programmes		‘I do think that if we do get things into a schedule, it generally happens’ (FG10) ‘Doing exercise regularly is the way to do it, having set times, doing it with other people…offering people a few things to do, at certain times allows people to plan week around that’ (FG12) ‘Having exercise built into hospital timetable and programme, exercise to attend, exercise on ward’ (FG15)								
Variety and innovation are needed		‘Link physical activity to what is happening in outside world for example when a bike race is on, we’ve had sort of a focus on some cycling’ (FG4) ‘I think that where possible, we have attempted to create some of that in terms of how diverse we make the rec hall timetable and the different options that you’re able to do’ (FG16)								
Flexibility and adaptation to patient preferences are essential		‘It comes down to personal choice, preferences are there, it’s going to change when people change, it shouldn’t be set in stone, so yes, you need a flexible model’ (FG5) ‘We’re always trying to gather what kind of activities they want to do’ (FG7) ‘Each patient is an individual, you can’t treat everyone the same, they have to have a buy-in’ (FG12)								
Motivational strategies and role modelling		‘If they get some initial benefits and start feeling it’s helping them, then the motivation does get a little bit contagious’ (FG4) ‘We kind of make a personalised programme and set some smaller goals and then they can review it each week and that really helps with motivation’ (FG6) ‘Staff participating with the patients…it’s a really good motivation’ (FG6)								

X, endorsed by at least one participant from forensic hospital in state/territory; –, no survey participants from the site endorsed this response.

Quantitative survey responses were integrated with qualitative focus group themes to provide contextual information and illustrate variability in practices across hospitals. Percentages indicate the proportion of survey participants reporting awareness of specific practices and are presented alongside thematic qualitative findings to augment interpretation. In Tables 3 to 5, a dash (–) indicates that no survey participants from a given site endorsed the response.

Our thematic analysis identified eight key themes: (a) lack of a standardised approach to physical health and activity; (b) inconsistent monitoring and documentation; (c) importance of general practitioners (GPs) and multidisciplinary teams (MDTs); (d) need for structured, adaptable programmes and engaged staff; (e) challenges related to patients, staff, ethics and infrastructure; (f) gaps in education, engagement and awareness; (g) need to balance nutrition and eating autonomy; and (h) importance of developing and implementing interventions.

### Lack of a standardised approach to physical health and activity

Table 2 presents the anthropometric, physiological, and lifestyle measures that participants reported as being recorded upon admission and during monitoring. The number (*n*) associated with each measure represents the number of respondents who indicated that the specific measure was conducted. Despite variability in responses from staff within the same facility, the data are still informative. The most common measures taken on admission and monitoring included blood pressure (admission: 68.2%, monitoring: 59.1%), resting heart rate (admission: 59.1%, monitoring: 45.5%), weight (admission: 59.1%, monitoring: 45.5%), height (admission: 54.5%, monitoring: 68.2%), body mass index (admission: 54.5%, monitoring: 31.8%) and full blood count (admission: 50%, monitoring: 31.8%). Two hospitals with exercise physiologists reported taking a physical activity history on admission. Additional tests such as vitamin B12, folate and strength assessments were also reported by a small number of participants. Survey and focus group data highlighted challenges in physical health assessments, including inconsistent responsibilities, variability in measures and limited use of fitness assessments. More than half of the participants were unaware of whether physical activity data were collected, although two hospitals used objective activity measures like the 6-min walk test, International Physical Activity Questionnaire and Simple Physical Activity Questionnaire, alongside other tests such as the Astrand Test and strength tests.

### Inconsistent monitoring and documentation

Table 3 presents findings on physical health monitoring practices. Staff from four hospitals reported recording metabolic monitoring data in both electronic and paper records, and staff from four hospitals also reported using a specific metabolic monitoring form, although only one participant indicated consistent use of this form.

Health monitoring frequency varied widely, with responses ranging from ‘unsure’ to specific time frames like ‘3–6 months’ or ‘monthly’. Some participants also used more general terms like ‘regularly’ or ‘on request’. Focus group discussions further revealed uncertainty about practices, inconsistent documentation and a lack of data system integration. Staff from Queensland, South Australia and Western Australia noted that regular data collection (e.g. body mass index) had discontinued, creating gaps in monitoring and evaluating patient health trends.

### The importance of GPs and MDTs

Survey responses outlining staff involvement in supporting physical health assessments and interventions across forensic hospitals are summarised in Table 4. There was variability noted across states/territories. A range of staff occupations were reported to provide support for physical health assessments, with psychiatrists, GPs and nursing staff being the most frequently endorsed roles. Physical health interventions saw broader participation from allied health professionals, with notable involvement from exercise physiologists, occupational therapists and physiotherapists in several states/territories.

Survey and focus group responses emphasised the collaborative efforts of the MDT in supporting patients’ physical health and activity. Allied health professionals, nursing staff and other team members work together to provide physical activities and support such as gym access, group activities and tailored exercise programmes. Access to GPs was viewed as essential for effective physical health monitoring. Exercise physiologists were seen as vital for tailoring physical activity programmes to patients’ needs. Physical health was discussed in MDT meetings, but its prioritisation varied. Although some teams included physical health as a key domain in care planning and reviewed it regularly, other teams reportedly did not prioritise it unless raised by specific MDT members.

### The need for structured, adaptable programmes and engaged staff

Table 5 outlines the physical activity environments and equipment available in forensic hospitals. All seven hospitals (*n* = 7) reported having access to a gym, and six provided outdoor exercise equipment. Less common facilities included tennis courts (*n* = 2) and swimming pools (*n* = 3). Other available equipment included stationary options such as treadmills (*n* = 6) and rowing machines (*n* = 5), as well as basketball courts, bicycles and weight machines.

A variety of individual and group programmes were offered, with all hospitals providing walking groups and volleyball. Six hospitals offered individual programmes and five provided group programmes, both on the ward and at the gym. Team sports like tennis and soccer, along with specialised activities such as badminton, yoga and water aerobics, were available in some settings.

Survey and focus group data emphasised the need for structured and adaptable activities that align with patient preferences to foster engagement. A mix of scheduled and spontaneous activities was seen as effective, with walking and volleyball being popular. Participants also highlighted the role of motivational strategies and role modelling in improving patient participation.

### Challenges related to patients, staff, ethics and infrastructure

Table 6 highlights the challenges of managing forensic patients’ physical health. Staff identified a range of patient-related barriers including low motivation, poor self-esteem, illness-related symptoms, body image concerns and gender issues.

**Table 6:** Staff perspectives on managing patients’ physical health and activity in forensic hospitals

Focus group results	Quotes
Theme: Challenges related to patients, staff, ethics and infrastructure	
Subthemes	Quotes – focus group (FG)
Patient-related barriers to engagement	‘Currently it’s lots of negative symptoms at the moment and low mood, low motivation’ (FG2) ‘I have women who opt out of ward exercise, especially opt out of the exercise group because they don’t feel comfortable being surrounded by men doing exercise’ (FG6)
Lack of staff, resources and infrastructure to support physical health initiatives	‘It’s not a matter of the service not wanting to give people those activities, it’s a matter of just literally having enough staff to do it, there’s…a bottleneck around staffing basically and that’s a chronic issue’ (FG5) ‘Our site is moving to a multi-story building…I do worry whether you know in the high floors how easy it will be for people to access the grounds’ (FG11)
Balancing patient autonomy with institutional policy and ethical considerations	‘One of the biggest struggles we have is weighing up patient’s autonomy, how much we insist people go, there’s some people who are reluctant to do anything…we can’t actually drag them out’ (FG4) ‘We would want to be structuring things and kind of mandating if you like, rules and models of what we want and then balancing that against patient choice, autonomy, right to self-determination’ (FG11)
Physical health and activity are often deprioritised in forensic hospitals	‘At the core of our service is risk, mental state, and exercise and physical health is pretty much down lower on the priority list’ (FG6) ‘You have a Care Pathway Team (CPT) meeting for each consumer…where you talk more broadly, discusses medical and mental health issues, but you do feel like physical health definitely takes a backseat’ (FG8)
Risk concerns affect assessment and physical activity opportunities	‘They have their physical…the work up done initially, the bloods, the measurements and so on but it’s more about containing the immediate risks’ (FG1) ‘Not allowed free weights, not allowed anything that could be used as a ligature… therabands need to be locked up’ (FG13) ‘Risk awareness seems to kill innovation’ (FG14)
Organisational barriers to integrating physical health into care	‘Some patients can’t be on campus at the same time as other patients and needs to sometimes be two staff if one staff member is away or held up, then they can’t have their access…there needs to be a bit more planning and thinking around how patients on the secure treatment orders get access to physical exercise’ (FG8) ‘Guidelines…very clearly say you should be offering lifestyle interventions…but in secure services, it’s split in terms of what the service’s agenda is’ (FG10)
Theme: Gaps in education, engagement and awareness	
Subthemes	Quotes – survey participant (P) and focus group (FG)
Challenges in delivering consistent and accessible physical health education	‘I provide education in my group programmes…I have also developed an information pamphlet for which I am seeking endorsement to provide to patients to outline the risks and what they can do about it’ (P10) ‘Treatment in a forensic unit is not usually a patient or carer decision, so providing this type of information may fuel feelings of disempowerment and non-engagement’ (P12) ‘Education is something…a lot of these people have never eaten a healthy diet… I think, we’ve got to approach it from an education side of things’ (FG4)
Limited patient and carer involvement in education and physical health initiatives	‘Patient groups are formed to assist in developing physical activity events for the forensic hospital …they are also consulted via the consumer advisory committee regarding all things physical health’ (P22) ‘Families involved in various aspects…but…never been involved in the physical health aspect of it’ (FG9)
There is a need to improve staff confidence in supporting physical health	‘We’ve got a schedule where we educate the registrars in terms of what’s due when’ (FG7) ‘We could probably have some procedure in in place and associated education and training to outline the importance of having regular metabolic monitoring’ (FG8) ‘It’s a real mixed bag in terms of how capable different staff feel working in that space’ (FG10)
Lack of awareness of physical activity guidelines and patient adherence among staff	‘Not aware of this or of the contents of the guidelines, except in a general sense, I am embarrassed to say’ (P6) ‘Unsure on specifics but significantly poor from observation’ (P18) ‘My estimation would be less than 30%’ (P22) ‘Not aware of any physical activity guidelines…not aware of any local guidelines’ (FG4)
Theme: The need to balance nutrition and autonomy	
Subthemes	Quotes – focus group (FG)
Food choice and institutional influence	‘We have very limited control over the food that our patients actually get here as well…the food that comes here is quite high in calories’ (FG6) ‘They get their fortnightly buys… they need to have a certain limit on unhealthy choices and then unlimited healthy choices… that is something that has changed over the years’ (FG6) ‘A lot of the leaves seem to be focused on food’ (FG7) ‘Organisation recognising need for role of dietitian and looking to increase…recognition from executive recognising that these positions are needed’ (FG13)
Role for education for staff and patients regarding healthy eating	‘Education is something…a lot of these people have never eaten a healthy diet, and they’re really attracted to the quick sugar, sugary food, high salt content, high fat content’ (FG4) ‘I did some training on eating disorders and really, really important not to demonise unhealthy foods’ (FG6) ‘There is also those kinds of educational sessions that will run throughout the year, and we get like patients’ involvement’ (FG6)
Shifting away from food-based incentives to alternative motivators	‘The difficulty is incentivising things…we’ve increasingly moved away from incentivising with food and junk food for obvious reasons, but there’s not that much that motivates people’ (FG4) ‘We’ve had to say to them…when you’re coming to community meeting, we shouldn’t really have to bribe people with biscuits to get them there’ (FG8)
Ethical concerns around food choices	‘You’re balancing wanting to foster autonomy against kind of wanting to rehabilitate someone to the best you can…when people will often love to buy some sugary chocolate…that is their decision…forensic patients have a very small amount of residual freedom’ (FG4) ‘Everyone has a human right to have nutritious food, and that’s why we’re involved in the food service menu’ (FG8) ‘That’s their choice in being able to access the kiosk, should never be determined by us’ (FG16)
Theme: The importance of developing and implementing interventions	
Subthemes	Quotes – focus group (FG)
Lessons to be learned from previous lifestyle interventions	‘We used Fitbits to track their activity…because we were supporting exercise sessions, their level of activity went up during the programme…and I think all of their metabolic ages went down and then we could track participation after the programme finished’ (FG9) ‘STRIDE [Self-directed Treatment for Obesity Reduction and Diabetes Education]…the way that the programme was structured was with behaviour change principles in mind, where each session is structured’ (FG10)
Planning and expanding future health and activity initiatives	‘Starting a physical and healthy eating group…doing some baseline measurements to see how much exercise people are doing in the unit and what kind of food people are eating’ (FG1) ‘HEAL [Healthy Eating Active Lifestyle]…it’s going to be piloted in rehab ward and then hopefully rolled out across the high secure units…it’s about healthy eating and the other half is doing physical exercise’ (FG3)

Lack of staff, resources and infrastructure to support physical health initiatives was a noted subtheme. Staffing shortages and limited access to GPs, dietitians, physiotherapists and exercise physiologists affect physical health initiatives. Infrastructure changes, such as a change to a multi-story building, may further restrict access to physical activities.

Another subtheme was the tension related to balancing patient autonomy with institutional policy and ethical considerations. Examples of concerns raised included restrictions on food at activities, balancing patient choice with structured exercise mandates and balancing patient autonomy with institutional policies.

Forensic hospitals face challenges in balancing mental and physical healthcare, with focus group participants noting that physical health and activity are often deprioritised with mental health and risk management takes precedence over physical health.

This challenge is further underscored by the impact of risk concerns on both assessment and physical activity opportunities. Safety issues have restricted access to outdoor activities and gyms, although limitations on equipment (e.g. free weights, therabands, and boxing) have reduced the range of physical activity options available.

Finally, organisational barriers, including scheduling conflicts, movement restrictions and delays in equipment repairs, further complicate the integration of physical activity into patient care.

### Gaps in education, engagement and awareness

Gaps in education, engagement and awareness hinder physical health promotion in forensic hospitals, as outlined in Table 6. Challenges include inconsistent and inaccessible physical health education for patients. Although some staff integrate education into group programmes and develop resources, there is no standardised approach. Staff noted that education should be ongoing. One participant noted that providing health information can sometimes disempower patients who have limited control over their treatment.

Patient and carer involvement in education and physical health initiatives was limited. Survey data showed that many participants did not provide information about obesity risks related to the forensic environment (40.9%). About half were unsure or provided no answer, whereas 31.8% reported offering general health education, including food choices and medication side-effects. Staff were more likely to discuss strategies for improving health outcomes (68.2%). Staff reported varying levels of confidence in supporting patients’ physical health, suggesting that there is a need to improve staff confidence. Implementing more structured education, targeted training and clear procedures such as those for metabolic monitoring and medication management were offered as strategies that could enhance staff competence and foster greater health promotion.

There was a lack of awareness of physical activity guidelines, and most survey participants were unsure about patient adherence to physical activity recommendations.

### The need to balance nutrition and eating autonomy

Staff consistently highlighted challenges related to nutrition and autonomy (Table 6). Issues around food choice and institutional influence highlighted the limited control staff had over food provision as well as the role of ‘buy-ups’ and the focus of leave being related to food. There was growing recognition of the need for dietitians to improve patient dietary care.

Education on healthy eating for both staff and patients was another subtheme. Staff valued prior training on using non-stigmatising language around food and suggested more individual and group discussions to improve food literacy.

A shift away from food-based incentives toward alternative motivators was also suggested. Staff noted that they had historically reinforced unhealthy eating habits by facilitating extra snack requests and allowing poor food choices, often as a way to compensate for patients’ restricted freedoms.

Balancing eating autonomy with dietary health posed ethical challenges for staff. Some staff argued that the right to eating autonomy must be balanced with the duty of care, as evidence shows that forensic patients often gain weight, which increases cardiometabolic risk and leads to poorer health outcomes.

### The importance of developing and implementing interventions

Staff reported limited trials of structured lifestyle interventions and emphasised the need for better implementation and evaluation (Table 6). Staff highlighted lessons from past programmes, noting challenges in execution and measuring effectiveness. Specific initiatives such as Group Occupation, Health, Exercise and Rehabilitation Treatment (GO HEART) and Self-directed Treatment for Obesity Reduction and Diabetes Education (STRIDE) were mentioned, along with a Fitbit-based intervention that successfully increased activity levels, reduced metabolic age and encouraged sustained gym participation.

Several forensic hospitals were expanding health and activity initiatives. The High Secure Inpatient Service in Queensland planned to pilot the Healthy Eating Active Lifestyle (HEAL) programme, integrating nutrition and exercise. The Dhulwa Secure Mental Health Unit in the Australian Capital Territory recently established a physical health group to develop new interventions, whereas the Forensic Hospital in New South Wales has established a metabolic clinic to support patients at risk of, or diagnosed with, metabolic syndrome.

## Discussion

Our findings contribute to the growing literature focussing on the health of forensic in-patients. From survey and focus group responses eight themes emerged: (a) lack of a standardised approach to physical health and activity; (b) inconsistent monitoring and documentation; (c) importance of GPs and MDTs; (d) need for structured, adaptable programmes and engaged staff; (e) challenges related to patients, staff, ethics and infrastructure; (f) gaps in education, engagement and awareness; (g) need to balance nutrition and eating autonomy; and (h) importance of developing and implementing interventions.

No forensic hospital had a clear, comprehensive approach to health assessment and monitoring. Assessments are challenging, as individuals are often severely unwell at admission, making physical examinations and blood tests difficult. Current recommendations include physical health screening as soon as feasible, ideally within 24 h of admission. Guidelines from New South Wales Health, the Royal Australian and New Zealand College of Psychiatrists and expert consensus statements outline minimum standards for screening, including body mass index, waist circumference, blood pressure, glucose regulation, fasting lipids and blood-borne viruses.^11,22,23^ Online survey results indicated these standards were not being met, with waist circumference monitoring reported in less than half of surveyed hospitals.

Staff from two forensic hospitals reported using a physical assessment template. Similarly, staff from four forensic hospitals noted the availability of a specific metabolic monitoring form, yet only one participant reported consistent use. Although adherence with metabolic monitoring has been studied in general psychiatric populations, no known research has focused specifically on forensic settings.^26,27^ Studies indicate that simple interventions, such as staff training and education, can improve monitoring rates.^28^ Some services recorded metabolic monitoring in both electronic records and paper charts, leading to duplication. Innovative technological solutions such as integrated platforms with automated reminders for due assessments and alerts for out-of-range measures could enhance adherence and streamline documentation.

A key finding of this research is the need for greater staff expertise. This aligns with recent calls from Dietitians Australia and Exercise and Sports Science Australia for expanded access to accredited exercise physiologists and practicing dietitians as part of comprehensive mental health treatment plans.^29^ Participant responses highlighted that services with exercise physiologists were better equipped to offer a broader range of physical activities and more personalised interventions. Recent research supports the role of exercise physiologists and dietitians in delivering lifestyle interventions for individuals with mental illness.^30,31^ Although exercise physiologists contribute specialised expertise in physical activity prescription within Australian forensic services, this professional role is not universally available across international settings. In other jurisdictions, similar functions may be undertaken by other health professional such as physiotherapists, occupational therapists or nursing staff with additional training in physical activity and health promotion. Regardless of professional background, the presence of staff with dedicated expertise in physical activity prescription appears critical to addressing the substantial physical health burden experienced by forensic in-patients.

A range of barriers to engagement in physical activity including patient-related factors (motivation, self-esteem and illness-related symptoms) were identified by staff. This is in line with patient perspectives who noted that lack of motivation and resourcing, staffing issues and problems with equipment hindered engagement.^18^ Forensic hospitals face challenges in prioritising physical health. Risk concerns affect physical activity initiatives, restricting outdoor and gym access, and limiting equipment options. Organisational barriers, such as scheduling conflicts, patient movement restrictions and delays in equipment repairs, further hinder the integration of physical activity into care.^17^

Concerns about human rights and patient autonomy in forensic mental health settings have been highlighted.^32,33^ These issues were raised by focus group participants, particularly regarding patient food choices, restrictions on kiosk access and participation in physical activity interventions. There is a growing shift away from using food as an incentive and toward implementing strategic, organisation-wide policies that balance patient autonomy with health promotion. Research on diet and nutrition within forensic populations remains limited, requiring services to rely on findings from general hospitalised populations.^34^ Efforts to modify food access must carefully consider ethical implications while ensuring that interventions are evidence-based and promote long-term health improvements.

Forensic hospitals should consider adapting evidence-based lifestyle interventions from general mental healthcare.^15^ For example, HEAL, part of the Australian Government’s Healthy Communities Initiative, has demonstrated significant benefits, including increased physical activity, reduced sitting time, improved diet and reductions in body mass index, waist circumference and blood pressure.^35^ Given the well-documented challenge of low motivation among individuals with mental illness, forensic hospitals must actively implement techniques such as positive encouragement to foster sustained participation.^36–38^ Autonomy-supportive approaches, which allow patients some control over their activity choices, have been shown to enhance motivation.^39,40^ The MDT plays an important role in fostering motivation through structured goal-setting, continuous support and reinforcing progress.

This study has several limitations. The online survey relied on self-reported data, making it susceptible to recall bias and participants’ reflective perceptions rather than verified site information. Responses to some survey questions showed substantial variability both within and across sites, particularly in relation to staffing levels, thereby limiting the reliability of quantitative findings for certain themes. Although 63 responses were missing across the 25 survey questions, analyses were based on the available data for each question. Given the presence of multiple respondents per site and the assumption that missing responses largely reflected individual knowledge gaps rather than systematic non-response, the impact of missing data on overall findings is likely minimal. In addition, participation was voluntary, and respondents may therefore not be fully representative of all forensic hospital staff. Although survey and focus group participants were broadly similar in professional roles, quantitative and qualitative data were collected from different individuals, which may have influenced the perspectives captured. Further, while the integration of survey and focus group findings enabled a more comprehensive interpretation of hospital practices, the combined presentation of quantitative data and qualitative themes reflects participants’ knowledge and perceptions rather than objective verification, and should be interpreted accordingly. Finally, the focus groups provided valuable qualitative insights, but they were also limited by sample size and may not have fully captured the diversity of experiences across forensic hospitals. This study did not include direct patient perspectives; however, patient views and experiences have been explored in prior research.^17,18^

Future research could focus on evaluating physical health monitoring practices in forensic hospitals, including the effectiveness of electronic health record integration in improving documentation, adherence and follow-up care. Assessing the impact of targeted training programmes on staff confidence, knowledge and adherence with physical health screening and intervention protocols could be another key area of inquiry. Longitudinal studies could explore the long-term health trajectories of forensic patients, comparing those who receive structured physical health interventions with those who do not, to assess their impact on health outcomes, lifestyle measures, morbidity and mortality. Comparative research across jurisdictions could help identify best practices and highlight areas for improvement, with the development of key performance indicators facilitating meaningful cross-site evaluations. Benchmarking of key performance indicators is promoted in the mental health field as a means of improving service quality.^41^ However, benchmarking physical health and activity has not yet formally been undertaken in Australia.

In conclusion, our study highlights the need for systemic reforms in forensic hospitals to ensure consistent physical health assessment and monitoring, better integration of multidisciplinary care and enhanced patient engagement. To successfully integrate improved physical health management into forensic hospitals, a collaborative and multifaceted approach is required, combining policy reform, resource investment, workforce training and patient-centred programme design. It is recommended that key performance indicators be developed to monitor service quality and outcomes, with the aim of enhancing patient care through increased accountability and a focus on continuous improvement.

## Supporting information

10.1192/bjo.2026.12013.sm001 Moss et al. supplementary material Moss et al. supplementary material

## Data Availability

The data-sets used and/or analysed during the current study are available from the corresponding author, K.M., on reasonable request.

## Supplementary material

The supplementary material is available online at https://doi.org/10.1192/bjo.2026.12013

## References

[1] Tully J , Hafferty J , Whiting D , Dean K , Fazel S. Forensic mental health: envisioning a more empirical future. Lancet Psychiatry 2024; 11: 934–42. 38945145 10.1016/S2215-0366(24)00164-0

[2] National Mental Health Commission. Monitoring the Performance of Australia’s Mental Health System: National Report Card 2023 . Australian Government, 2023 (https://www.mentalhealthcommission.gov.au/sites/default/files/2024-07/national-report-card-2023_0_0.pdf [cited 20 Mar 2025]).

[3] Australian Human Rights Commission. A Human Rights Act for Australia. Australian Human Rights Commission, 2022 (https://humanrights.gov.au/sites/default/files/free_equal_hra_2022_-_main_report_rgb_0_0.pdf [cited 8 Aug 2025]).

[4] Roberts R , Wong A , Lawn S , Lawrence D , Johnson C. Mortality of People Using Australian Government-Funded Mental Health Services and Prescription Medications: Analysis of 2016 Census, Death Registry, MBS and PBS Data. Charles Sturt University, 2024 (https://researchoutput.csu.edu.au/en/publications/mortality-of-people-using-australian-government-funded-mental-hea [cited 22 Jun 2025]).

[5] Lawrence D , Hancock KJ , Kisely S. The gap in life expectancy from preventable physical illness in psychiatric patients in Western Australia: retrospective analysis of population-based registers. BMJ 2013; 346: f2539. 23694688 10.1136/bmj.f2539 PMC3660620

[6] Andersson P , von Schreeb A , Johansson L , Sturidsson K , Wetterborg D , Sorjonen K. Changes in body mass index during mandatory forensic psychiatric care: findings from a long-term (2009–2020) cohort study based on swedish registry data. Int J Forensic Ment Health 2024; 23: 106–16.

[7] Jeandarme I , Goktas G , Claessens B , Michem T , Van Poucke S , Verbeke G. Smoking, obesity, and metabolic syndrome in two high security settings. Int J Forensic Ment Health 2024; 23: 499–513.

[8] Moss K , Meurk C , Steele ML , Heffernan E. Physical health and activity of inpatients under forensic mental health care: a cross-sectional survey and audit of patients in a high secure setting in Queensland, Australia. Int J Forensic Ment Health 2024; 23: 12–23.

[9] Druss BG , Zhao L , Von Esenwein S , Morrato EH , Marcus SC. Understanding excess mortality in persons with mental illness: 17-year follow up of a nationally representative US survey. Med Care 2011; 49: 599–604. 21577183 10.1097/MLR.0b013e31820bf86e

[10] Rees C , Thomson L. Exploration of morbidity, suicide and all-cause mortality in a Scottish forensic cohort over 20 years. BJPsych Open 2020; 6: e62. 32552922 10.1192/bjo.2020.40 PMC7345667

[11] New South Wales Ministry of Health. Physical Health Care for People Living with Mental Health Issues: GL2021_006. New South Wales Ministry of Health, 2021 (https://www1.health.nsw.gov.au/pds/ActivePDSDocuments/GL2021_006.pdf [cited 22 Jun 2025]).

[12] Perry BI , Holt RIG , Chew-Graham CA , Tiffin E , French P , Pratt P , et al. Positive Cardiometabolic Health Resource (2023 Update). Royal College of Psychiatrists, 2023 (https://www.rcpsych.ac.uk/docs/default-source/improving-care/ccqi/national-clinical-audits/ncap-library/eip-2024/ncap-lester-tool-intervention-framework.pdf?sfvrsn=21e45dbd_17 [cited 22 Jun 2025]).

[13] Martland R , Teasdale S , Murray RM , Gardner-Sood P , Smith S , Ismail K , et al. Dietary intake, physical activity and sedentary behaviour patterns in a sample with established psychosis and associations with mental health symptomatology. Psychol Med 2023; 53: 1565–75. 34420532 10.1017/S0033291721003147 PMC10009388

[14] Deenik J , Tenback DE , Tak ECPM , Rutters F , Hendriksen IJM , van Harten PN. Changes in physical and psychiatric health after a multidisciplinary lifestyle enhancing treatment for inpatients with severe mental illness: the MULTI study I. Schizophr Res 2018; 204: 360–7. 30055884 10.1016/j.schres.2018.07.033

[15] Moss K , Meurk C , Steele ML , Heffernan E. The physical health and activity of patients under forensic psychiatric care: a scoping review. Int J Forensic Ment Health 2022; 21: 194–209.

[16] Rogers E , Kinnafick F-E , Papathomas A. Physical activity in secure settings: a scoping review of methods, theory and practice. Ment Health Phys Act 2019; 16: 80–95.

[17] Anthony J , Papathomas A , Annandale A , Breen K , Kinnafick FE. Experiences of physical activity for adolescents in secure psychiatric care: staff and patient perspectives. Ment Health Phys Act 2023; 24: 100506.

[18] Moss K , Meurk C , Steele ML , Heffernan E. Physical activity of inpatients under forensic mental health care: a mixed methods study of patient knowledge, preferences, practices and identified barriers. Int J Forensic Ment Health 2025; 24: 119–31.

[19] Australian Government Department of Health. Prevention Compassion Care: National Mental Health and Suicide Prevention Plan. Australian Government Department of Health, 2021 (https://www.health.gov.au/sites/default/files/documents/2021/05/the-australian-government-s-national-mental-health-and-suicide-prevention-plan-national-mental-health-and-suicide-prevention-plan.pdf [cited 22 Jun 2025]).

[20] Queensland Health. *Better Care Together: A Plan for Queensland’s State-funded Mental Health, Alcohol and Other Drug Services to* 2027. Queensland Health, 2025 (https://www.health.qld.gov.au/__data/assets/pdf_file/0032/1178744/BetterCareTogether_HR.pdf [cited 22 Jun 2025]).

[21] O’Brien BC , Harris IB , Beckman TJ , Reed DA , Cook DA. Standards for reporting qualitative research: a synthesis of recommendations. Acad Med 2014; 89: 1245–51. 24979285 10.1097/ACM.0000000000000388

[22] Galletly C , Castle D , Dark F , Humberston V , Jablensky A , Killackey E , et al. Royal Australian and New Zealand College of Psychiatrists clinical practice guidelines for the management of schizophrenia and related disorders. Aust N Z J Psychiatry 2016; 50: 410–72. 27106681 10.1177/0004867416641195

[23] Lambert TJ , Reavley NJ , Jorm AF , Oakley Browne MA. Royal Australian and New Zealand College of Psychiatrists expert consensus statement for the treatment, management and monitoring of the physical health of people with an enduring psychotic illness. Aust N Z J Psychiatry 2017; 51: 332–7. 10.1177/0004867416686693 28343435

[24] Leech NL , Onwuegbuzie AJ. A typology of mixed methods research designs. Qual Quant 2009; 43: 265–75.

[25] Gale NK , Heath G , Cameron E , Rashid S , Redwood S. Using the framework method for the analysis of qualitative data in multi-disciplinary health research. BMC Med Res Methodol 2013; 117: 13. 10.1186/1471-2288-13-117 PMC3848812 24047204

[26] Benson C , Kisely S , Korman N , Moss K. Compliance of metabolic monitoring at rehabilitation facilities. Australas Psychiatry 2018; 26: 41–6. 29087209 10.1177/1039856217737899

[27] Tso G , Kumar P , Jayasooriya T , Kisely S , Siskind D. Metabolic monitoring and management among clozapine users. Australas Psychiatry 2017; 25: 48–52. 27590080 10.1177/1039856216665282

[28] Michael S , MacDonald K. Improving rates of metabolic monitoring on an inpatient psychiatric ward. BMJ Open Qual 2020; 9: e000748. 10.1136/bmjoq-2019-000748 PMC7375397 32699081

[29] Dietitians Australia. Australian First General Practitioner, Exercise Physiologist, and Dietitian Support Program Improves Well-being of Complex Mental Health Patients. Dietitians Australia, 2024 (https://dietitiansaustralia.org.au/sites/default/files/2024-05/202405%20-%20Australian%20first%20General%20Practitioner%20Exercise%20Physiologist%20and%20Dietitian%20support%20program%20improves%20wellbeing%20of%20complex%20mental%20health%20patients%20-%20Media%20Release.pdf [cited 20 Mar 2025]).

[30] Korman N , Fox H , Skinner T , Dodd C , Suetani S , Chapman J , et al. Feasibility and acceptability of a student-led lifestyle (diet and exercise) intervention within a residential rehabilitation setting for people with severe mental illness, GO HEART (Group Occupation, Health, Exercise And Rehabilitation Treatment). Front Psychiatry 2020; 11: 319. 32411024 10.3389/fpsyt.2020.00319 PMC7198865

[31] Wynaden D , Barr L , Omari O , Fulton A. Evaluation of service users’ experiences of participating in an exercise programme at the Western Australian State Forensic Mental Health Services. Int J Ment Health Nurs 2012; 21: 229–35. 22533330 10.1111/j.1447-0349.2011.00787.x

[32] Johnson M , Day M , Moholkar R , Gilluley P , Goyder E. Tackling obesity in mental health secure units: a mixed method synthesis of available evidence. BJPsych Open 2018; 4: 294–301. 30083382 10.1192/bjo.2018.26 PMC6066985

[33] Markham S. The need for practicable normative right-based social work practice in secure and forensic mental health services. Br J Soc Work 2023; 53: 1726–34.

[34] Osman NS , Md Nor N , Md Sharif MS , Hamid SBA , Rahamat S. Hospital food service strategies to improve food intakes among inpatients: a systematic review. Nutrients 2021; 13: 3649. 34684649 10.3390/nu13103649 PMC8537902

[35] Hetherington SA , Borodzicz JA , Shing CM. Assessing the real-world effectiveness of the Healthy Eating Activity and Lifestyle (HEAL) program. Health Promot J Austr 2015; 26: 93–8. 25903114 10.1071/HE14031

[36] Bacon N , Farnworth L , Boyd R. The use of the Wii Fit in forensic mental health: exercise for people at risk of obesity. Br J Occup Ther 2012; 75: 61–8.

[37] Tetlie T , Heimsnes MC , Almvik R. Using exercise to treat patients with severe mental illness: how and why? J Psychosoc Nurs Ment Health Serv 2009; 47: 33–40. 10.3928/02793695-20090201-14 19266974

[38] Verhaeghe J , De Maeseneer J , Maes L , Van Heeringen C , Annemans L. Health promotion in mental health care: perceptions from patients and mental health nurses. J Clin Nurs 2013; 22: 1569–78. 23294398 10.1111/jocn.12076

[39] Beebe LH , Smith K , Burk R , McIntyre K , Dessieux O , Tavakoli A , et al. Effect of a motivational intervention on exercise behavior in persons with schizophrenia spectrum disorders. Community Ment Health J 2011; 47: 628–36. 21113661 10.1007/s10597-010-9363-8 PMC3135691

[40] Vancampfort D , Stubbs B , Venigalla SK , Probst M. Adopting and maintaining physical activity behaviours in people with severe mental illness: the importance of autonomous motivation. Prev Med 2015; 81: 216–20. 26386141 10.1016/j.ypmed.2015.09.006

[41] Meehan TJ , Stedman TJ , Neuendorf KE , Francisco I , Neilson MG. Benchmarking Australia’s mental health services: is it possible and useful? Aust Health Rev 2007; 31: 623–7. 17973621 10.1071/ah070623

