# Supplementary material for: Physical health and activity management in forensic mental healthcare: hospital practices and staff insights from an Australian national study
Source: BJPsych Open. 2026 Jun 2;12(4):e153. doi: 10.1192/bjo.2026.12013 (PMC13237119; doi:10.1192/bjo.2026.12013)
Supplement: Moss et al. supplementary material [file S2056472426120134sup001.docx]

**Supplementary Material**

**Online Survey via Checkbox**

Demographics

1. State or Territory
2. Current role
3. How long have you worked in forensic services?

Physical Heath Initial Assessment:

1. What anthropometric and physiological health measures does your service gather on initial assessment?

- Weight
- Height
- Calculated BMI
- Waist circumference
- Blood pressure
- Resting heart rate
- ECG
- Diabetes Mellitus
- HbA1c
- Fasting glucose
- 2 random blood sugar levels
- Triglycerides
- HDL-C
- LDL-C
- Cholesterol
- Fasting insulin
- FBC
- eLFT
- TFT
- Hepatitis and HIV serology
- Smoking status
- Diet
- Exercise history
- Other: ­­­­­­­­­­­___________________________________________________________

1. Where are these measures recorded?

- Electronic form
- Paper form in patient’s chart
- Other: ___________________________________________________________

Physical Health Monitoring

1. What measures do you regularly collect for monitoring?

- Weight
- Height
- Calculated BMI
- Waist circumference
- Blood pressure
- Resting heart rate
- ECG
- Diabetes Mellitus
- HbA1c
- Fasting glucose
- 2 random blood sugar levels
- Triglycerides
- HDL-C
- LDL - C
- Cholesterol
- Fasting insulin
- FBC
- eLFT
- TFT
- Hepatitis and HIV serology
- Smoking status
- Diet
- Exercise history
- Other: ___________________________________________________________

1. How frequently are these physical health measures taken?
2. Where are these measures recorded?

- Electronic form
  - Does this form allow you to compare data?
- Paper form in patient’s chart
- Other: ___________________________________________________________

1. Do you have a specific Metabolic Monitoring form?
   - Is this form used consistently by staff?
2. How do you ensure that any physical health concerns are addressed? Do you have a care plan that includes physical health and activity?
3. Has your service collated any physical health data with regards to individuals under compulsory forensic psychiatric care?
4. How does your service ensure that an individual’s physical health needs are regularly assessed?

Physical Activity Measures

1. What physical activity measures does your service collect?

- Accelerometry
- 6-minute walk test
- VO_2max_
- International Physical Activity Questionnaire (IPAQ)
- International Physical Activity Questionnaire – Short Form (IPAQ-SF)
- Simple Physical Activity Questionnaire (SIMPAQ)
- Community Health Activities Model Program for Seniors (CHAMPS)
- Past Week Activity Questionnaire (PWA)
- Blair 7-day recall (7DR)
- Yale Physical Activity Scale (YPAS)
- Total Activity Measure 2 (TAMII)
- Australia Active Scale (AAS)
- 3-Month Physical Activity Checklist (3MPAC)
- Godin Physical Activity Questionnaire

Other______________________

1. Do you regularly update these measures?
2. Has your service collated any physical activity data with regards to individuals under compulsory forensic psychiatric care?
3. Are you aware of how many patients currently adhere to the World Health Organisation physical activity guidelines?

Staffing

1. What staff provide support for physical health assessment?

- General Practitioner
- Psychiatrist
- Nursing Staff
- Other____________________________________________________________

1. What staff provide support for physical health interventions?

- General Practitioner
- Psychiatrist
- Nursing Staff
- Physiotherapist
- Occupational Therapist
- Exercise Physiologist
- Personal Trainer
- Diversional Therapist
- Recreational Officer
- Other_________________________________________________________

Current staffing:

1. Do you have access to a General Practitioner at your site?

What is the equivalent FTE of the position/s?

What is the current FTE provided?

1. Do you have access to a Physiotherapist at your site?

What is the equivalent FTE of the position/s?

What is the current FTE provided?

1. Do you have access to an Occupational Therapist at your site?

What is the equivalent FTE of the position/s?

What is the current FTE provided?

1. Do you have access to an Exercise Physiologist at your site?

What is the equivalent FTE of the position/s?

What is the current FTE provided?

1. Do you have access to a Personal Trainer at your site?

What is the equivalent FTE of the position/s?

What is the current FTE provided?

1. Do you have access to a Diversional Therapist at your site?

What is the equivalent FTE of the position/s?

What is the current FTE provided?

1. Do you have access to a Recreational Officer at your site?

What is the equivalent FTE of the position/s?

What is the current FTE provided?

1. Is there anyone else employed at your site that supports the physical health and activity of inpatients under forensic mental health care?

Environment

1. Does your service have a suitable room for physical examination?
2. Which of the following are available to you?

- Tape measures
- Digital scales
- Height measures (calibrated rigid stadiometer)
- Glucometer and accessories
- Stethoscope
- Sphygmomanometer
- Urine analysis kit (dip stick type to check for microscopic haematuria, proteinuria and infection)
- Hand washing facility
- Disposable/surgical gloves
- Appropriate bio-hazard disposable units
- Privacy gowns and drapes
- Access to pathology order forms
- Access to online results

1. What spaces/equipment are provided for patients for physical activity?

- Gym
- Outdoor exercise equipment
- Tennis court
- Swimming pool
- Equipment on wards
  - Treadmill
  - Rowing Machine
  - Other
- Other____________________________________________________________

Physical Activity Interventions

1. What physical activity options are offered to patients?

- Individual program on ward
- Individual program at gym
- Group program on ward
- Group program at gym
- Swimming
- Tennis
- Soccer
- Volleyball
- Walking group
- Other____________________________________________________________

Consumer Information and Input

1. Do you provide access to information for patients and their carers about the potential for forensic hospital environments to cause obesity?
2. Do you involve patients in the development of strategies to improve physical health and activity outcomes at your service?

**Focus Group Interview Guide**

How does your service assess and manage physical health issues for patients under inpatient forensic psychiatric care?

Check summary data obtained from online survey. Are there any further details not previously covered?

Do you collect non-modifiable risk factors for cardiovascular disease e.g. family history? Where do you record this?

Are you aware of the Royal Australian and New Zealand College of Psychiatrists position statement on physical health? Does your service adhere to these guidelines?

How does your service ensure that patients meet the World Health Organisation physical activity guidelines?

What works well in your environment to support patients’ physical activity needs?

Does the service work with local health professionals, services and other organisations to increase its capacity to deliver and promote physical activity initiatives?

Is there a documented management plan to assist with patients’ physical health and activity? Who completes this? Where is it recorded?

Do you engage the individual and carers in strategies to ensure healthy living (e.g. diet and exercise)? How do you do this?

How do you address the risks of metabolic complications in treatment (e.g. do you consider switching to a weight-neutral antipsychotic agent for individuals)?

Do you use adjunctive measures to address weight gain in individuals on antipsychotic medications (e.g. metformin)?

What other professionals do you liaise with (e.g. endocrinology specialists)?

What (if any) evidence-based programs do you provide to address obesity and lack of exercise?

Has your service implemented any physical health or activity interventions? What did they involve? What were the results?

What challenges do you face in supporting the physical health and activity of patients under inpatient forensic psychiatric care?
